# Supplementary material for: Quantification of Wnt3a, Wnt5a and Wnt16 Binding to Multiple Frizzleds Under Physiological Conditions Using NanoBit/BRET
Source: Cells. 2025 May 30;14(11):810. doi: 10.3390/cells14110810 (PMC12153800; doi:10.3390/cells14110810)
Supplement: Supplementary file 1 [file cells-14-00810-s001.zip › cells-3610935-supplementary.pdf]

Supplementary Figure S1

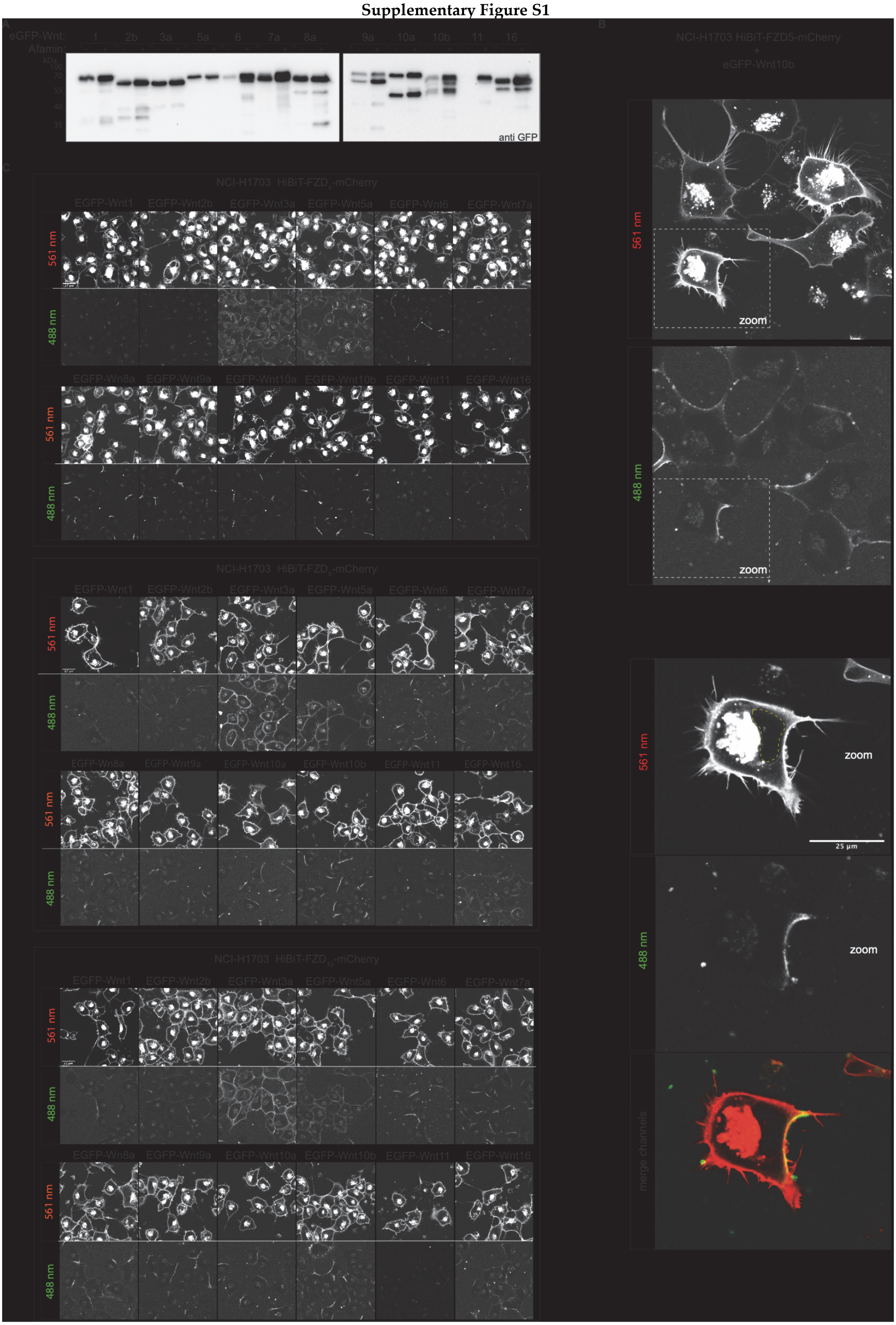

Supplementary Figure S1. Generation and testing of eGFP-Wnt fusion proteins. A) Western Blot (WB) of conditioned medium (CM) harvested from HEK293T cells transfected with the indicated constructs, confirming the secretion of full length eGFP-Wnt proteins with/without Afamin co-expression. **B)** Laser scanning confocal microscopy images of NCI-H1703 cells with stable integration of mouse HiBiT-FZD$_5$-mCherry, incubated for 3 h with eGFP-Wnt10b CM derived from Expi293™ suspension cells. The three lower images represent zoomed-in images of boxed areas shown in the upper images and highlight the asymmetric nature of how some Wnt's appear to associate to cells. **C)** Laser scanning confocal microscopy images of NCI-H1703 cells with stable integration of mouse HiBiT-FZD$_{4,8,10}$-mCherry, incubated for 3 h with the indicated eGFP-Wnt CM derived from Expi293™ suspension cells.

Supplementary Figure S2

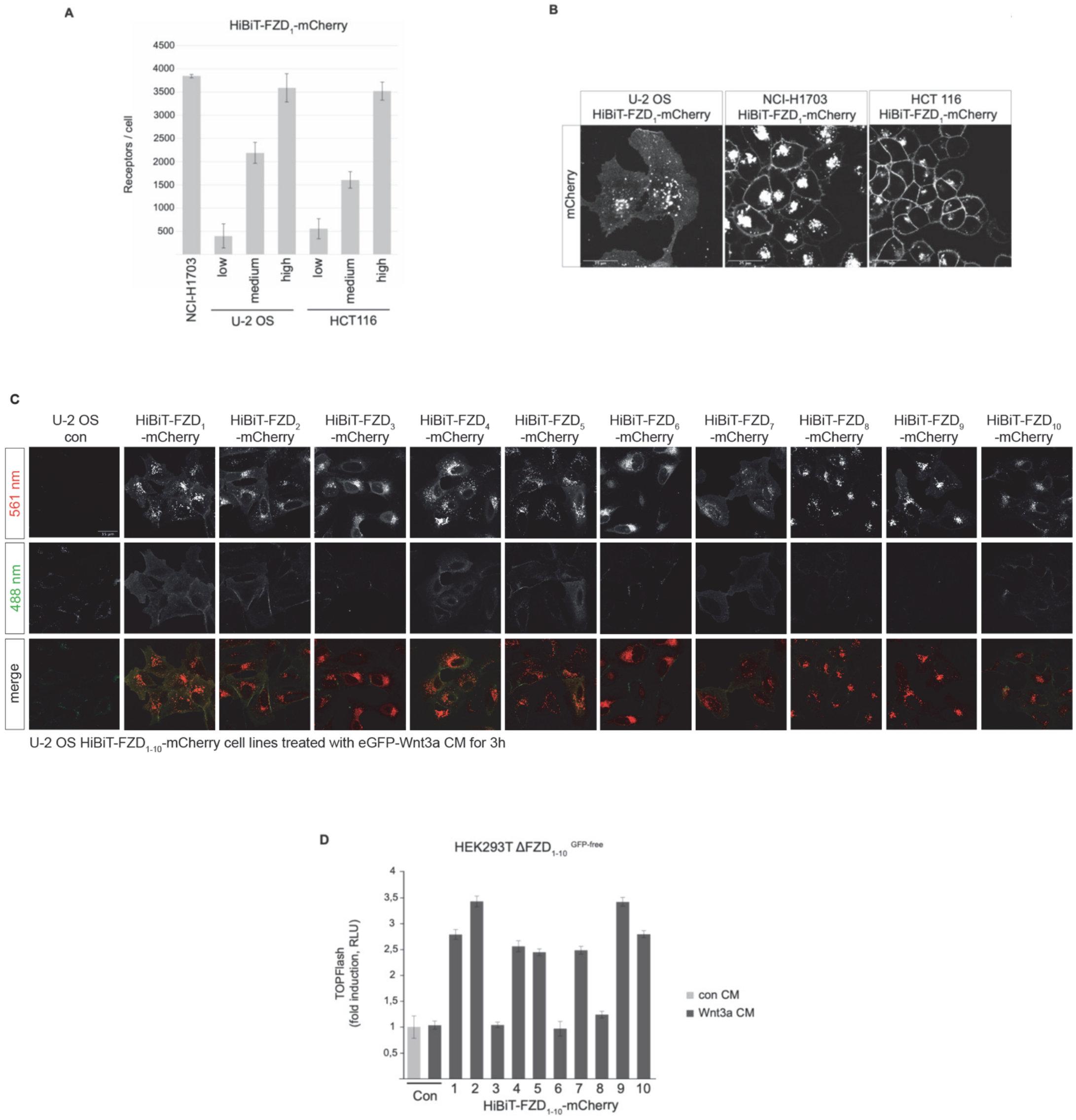

Supplementary Figure S2. Comparison of stable expression levels of HiBiT-FZD$_1$ in NCI-1703 U-2 OS and HCT116 cells. A) Indirect quantification of HiBiT-FZD$_1$-mCherry at the cell surface of the indicated FACS sorted stable cell lines. **B**) Lazer scanning confocal microscopy images comparing the expression of HiBiT-FZD$_1$-mCherry in the indicated FACS sorted cells. **C**) Laser scanning confocal microscopy images of living U-2 OS cells with stable integration of mouse HiBiT-FZD-mCherry constructs as indicated. Cells were incubated for 3 h with eGFP-Wnt3a condition medium (CM) derived from Expi293™ suspension cells. D) TOPFlash reporter assay in ΔFZD$_{1\text{-}10}^{GFP\text{-}free}$ HEK293 cells, showing the relative Wnt/β-catenin signaling activity of the indicated mouse HiBiT-FZD-mCherry receptors. Error bars represent ± S.D. form means of 4 independent biological samples.

Supplementary Figure S3

A

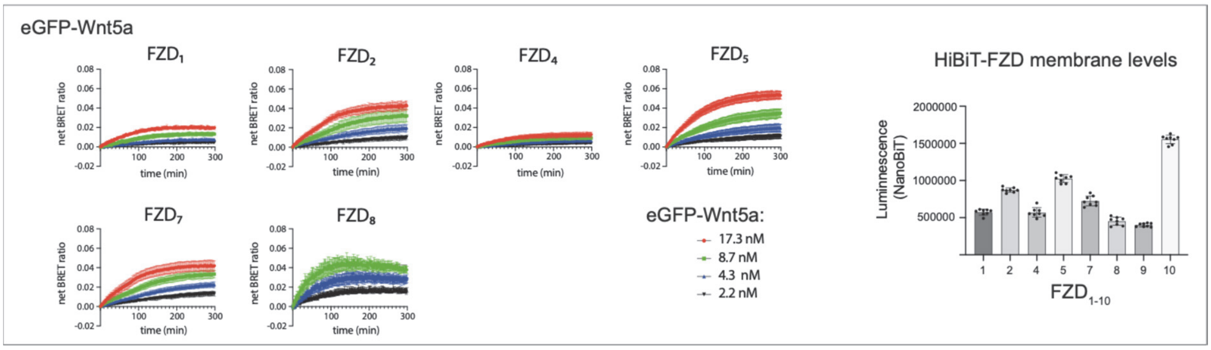

B

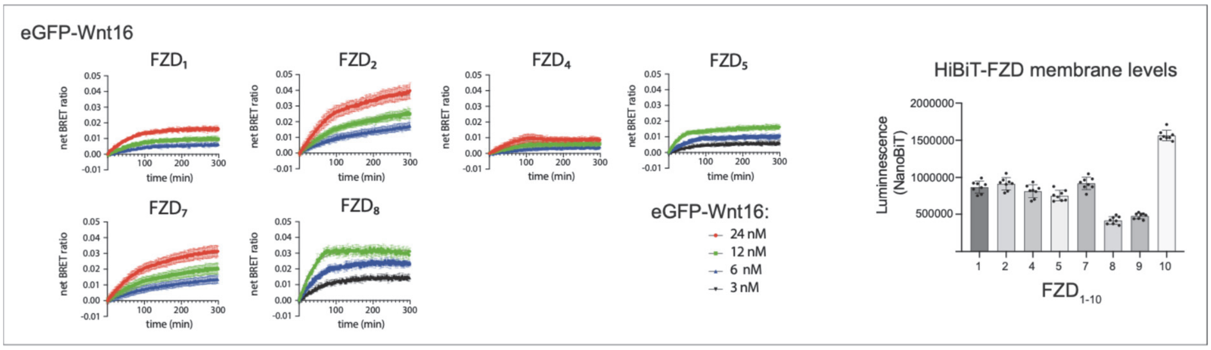

C

	statistic	p. value	parameter	method
eGFP-Wnt3a	18,3723	0,0054	6	Kruskal-Wallis rank sum test
eGFP-Wnt5a	18,5014	0,0024	5	Kruskal-Wallis rank sum test
eGFP-Wnt16	12,9452	0,024	5	Kruskal-Wallis rank sum test

Supplementary Figure S3. Comparison of Wnt5a and Wnt16 binding to multiple FZD expressing cell lines. Association kinetics of eGFP-Wnt5a (**A**) and eGFP-Wnt16 (**B**) with U-2 OS cells expressing the indicated HiBiT-FZD proteins. Binding curves were generated over time using 2.2, 4.3, 8.7, 17.3 nM of eGFP-Wnt5a or 3, 6, 12, 24 nM of eGFP-Wnt16. BRET ratios were measured every 80 s for a total of 5 h. Raw data were fitted to the 'two or more hot concentrations model' and are presented as mean ± SEM form n= 3-4 individual experiments. Estimation of relative HiBiT-FZD cell surface levels in FACS-sorted U-2 OS cells used for each assay are shown in graps on right. C) The open source software R was used for Kruskal-Wallis rank sum test statistics to determine significance between different FZD samples within experiments.

Supplementary Figure S4

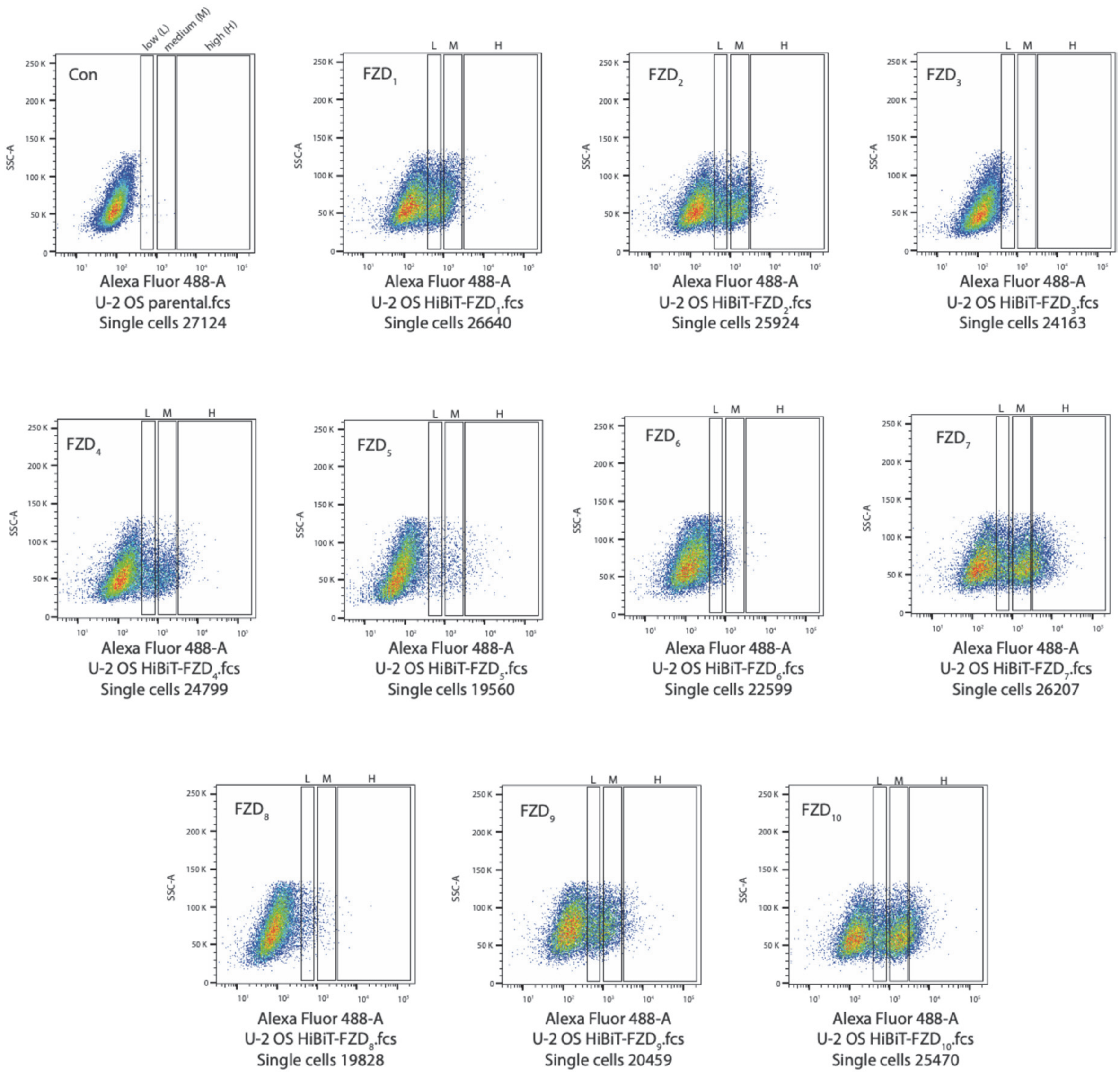

Supplementary Figure S4. FACS sorting of U-2 OS cells stably expressing HiBiT-FZD$_{1-10}$. Single cell sorting of U-2 OS cells stably expressing HiBiT-FZD-mCherry at the cell surface. U-2 OS cell suspensions were incubated with mouse anti-HiBiT antibody following by labelling with anti-mouse Alexa 488 antibody. All cells were analyzed on one dimensional plots of the green channel- 488 nm for HiBiT positive cells. Gated in black as low (L), medium (M) and high (H) are cell populations with di erent expression levels of HiBiT-FZD at the cell surface. Parental U-2 OS cells were used as background control. Cells gated for low, medium and high were single cell sorted in 96 well plate.
